# Renal Decapsulation for Puerperal Eclampsia1Read at the forty-first annual meeting of the Medical Association of Central New York, held at Buffalo, October 27, 1908.

**Published:** 1909-01

**Authors:** William B. Jones

**Affiliations:** Rochester, N. Y.


### Renal Decapsulation for Puerperal Eclampsia.[1]

BY WILLIAM B. JONES, M. D., Rochester, N. Y.

ECLAMPSIA occurs in about four of one thousand labors and one of these is fatal. This varies greatly in limited numbers of cases, periods of time, and locations, but is very constant in the statistics of many thousand when combined. Those women who engage and report to the physician during preg-

1. Read at the forty-first annual meeting of the Medical Association of Central New York, held at Buffalo, October 27, 1908.

nancy are treated on appearance of premonitory symptoms, and it is prevented in most, but not in all patients. Some women do not and then we have no chance to employ prophylaxis. When eclampsia occurs treatment saves three, but after all methods have been exhausted one patient is still growing worse, and there is nothing more to do but continue some of the same remedies which have not prevented rapid increase of her danger, and she dies. Just here renal decapsulation offers to save. It was first proposed by Dr. George M. Edebohls of New York, as is well known.

I have secured abstracts of all cases published in medical literature up to September 20, 1908. They are twenty-four and to add two of my own, twenty-six in all. In every one, other measures had been exhausted and the patient was growing worse, there had been coma for hours, most of them had fifteen or more convulsions, rapidly increasing fever and weak rapid pulse. Some had pulmonary edema very marked. In other words, all were in a condition threatening speedy dissolution. Every autopsy showed rapid extreme parenchymatous degeneration, comparable to what might be caused by a most violent poison, complete strangulation of blood supply or violent infection, and all who recovered showed in the urine evidence of a severe acute nephritis.

Of the recoveries the one with the mildest symptoms had about fourteen convulsions in twelve and a half hours, during which delivery was instrumental; operation at 7 p. m., two more convulsions, but next morning the patient was bright and well started in convalescence. There were also among the recoveries almost all kinds of cases,—before term with premature labor, before term operation and pregnancy continuing to a normal labor at term, full term with unassisted delivery, with ordinary forceps delivery and with forced delivery. One of the worst cases that recovered had the first convulsion six hours after a severe labor, did not recover consciousness but passed into complete coma and so remained. After twelve hours she had had fourteen convulsions with progressive rise of temperature to 105, weakening and rapidity of pulse till it was about 200, but could not be accurately counted, failure of circulation until the surface was livid and change of color upon removal of pressure of a finger tip was very slow, pulmonary edema until the fluid ran from the mouth in a continuous stream for some time after turning the patient on to her face, suppression of urine to the extent of only two ounces by catheter in six hours or longer,—altogether a typical moribund condition. Yet this woman in the second twenty-four hours after operation passed over 100 ounces of urine, besides what was passed with about eight stools which were also mostly water. This urine contained a very large amount of albumin and many kinds of casts in abundance. Her recovery was rapid and now after eighteen months she is perfectly well. The urine is normal. Another, one of Dr. Edebohls's cases, had typhoid in the fourth month, nephritis beginning in the seventh. There were convulsions at the end of the eighth, five severe ones in sixteen hours. After forced delivery she was free from them forty-six hours, but had six or more during the next eighteen. Then she had double *decapsulation,* no more eclampsia and rapid recovery. I am aware that desperate cases may recover without renal decapsulation but this one was extreme. I would like to know of all as bad that have.

The theory of the action of this operation is reasonable. Whatever the remote cause of eclampsia the condition is mainly toxemia and insufficient elimination. This is shown by its mode of onset, the prodromata when present, and by the degenerations of parenchyma of liver and kidneys, as well as by the improvement from eliminative treatment of any kind when the condition has not gone too far. One might suppose that elimination by the kidneys is normal if urine of ordinary density and quantity is being passed, but that does not matter because far more than normal elimination is demanded and there is no assurance that what would ordinarily be sufficient is so at the time. Every one who recovered after operation, very soon began to pass largely increased amounts of urine and solids, of urea, too, when that was measured.

The kidneys are particularly affected. They become congested to the extent of stoppage of function and later total disorganisation. Irritated by toxins in the urine the parenchyma begins to swell, the finest vessels are compressed and cannot carry away the blood brought into the organ, congestion of irritation is increased by congestion of mechanical obstruction and passes on to degeneration of parenchyma, which is practically necrosis, but not quite as rapid as it would be if the circulation were entirely blocked as by a ligature around the main vessels. This is not theory devised to fit the symptoms or even the post mortem findings, but an explanation of conditions at operations upon those kidneys during the progress of the disease, observations upon the process itself, not upon the results after all damage has been done.

All operations done fairly early showed enormous swelling, in some described in apparently extravagant terms; several in only one kidney, some in both, the bulk of the organ as a whole being greatly increased. Those done late showed the same with softening that in one or two approached putrefaction which did occur very quickly after death. All were darkly colored by congestion, the most so being almost black. Hemorrhagic infarcts and large hematomata have been found. It should be noted that one kidney may seem to be nearly or even quite normal, the other showing all the disease.

Division of the capsule simply relieves pressure and permits the compressed vessels to carry blood again. If a sponge be squeezed in the hand water cannot pass through it, but if the hand is opened it swells. its channels open and permit the passage of a good stream. During the operation there is also some bleeding from the kidneys which helps and it may be well to favor that by small incisions. The patient is usually comatose and needs no anesthetic. She should be placed prone upon a table with a kidney pillow under her waist. Make any usual incision to reach the kidney; part the perirenal tissues enough to see half its length, cut a line longidutinally, near but necessarily immediately upon the convex border. slip two fingers inside the capsule and strip it off the kidney. Tuck it back so that it will stay off from the raw cortex and suture the loin incision rapidly. One minute should complete each side except closing the incision.

There need be no hesitation in deciding whether an operation is advisable, or when. The first twelve hours of eclampsia usually decides a woman's fate. The danger becomes greater with the frequency and severity of the convulsions, the persistency and depth of coma, rapidity and weakness of pulse, the height above normal of temperature and the rapidity of its rise, with the quantity of albumin and casts and inversely with the quantity of urine. Pulmonary edema denotes the beginning of death, but not always that it is too late to bring back life. If at the end of twelve hours she has had fifteen or more convulsions which are growing more severe or often, coma is uninterrupted, temperature is rising and pulse growing faster and weaker, she is doomed unless more can be done than remedies already used can do.

Before that time the physician should know whether non-surgical treatment will cure the patient. By rapid intelligent work all methods should be applied, and before eight hours he will have used them and secured results that either justify continuing in the same way or demonstrate that they are unequal to the case. As soon as that point is reached renal decapsulation is indicated immediately and cannot be hastened too much. The changes in the kidneys progress so rapidly that the condition is an emergency and should be treated like one, with uninterrupted presence of the physician, exhibition of remedies, rapidly, systematically without procrastination. Let him know what he will and will not do, and act promptly every move.

I can think of no possible objection to decapsulation. There is no anesthetic or shock and other treatment is continued without interruption. Of the twenty-six cases now known, all had reached a condition believed to be hopeless. Many, as is true of every measure not yet established in general confidence, were delayed long beyond the proper time, one had convulsions, coma, jaundice and anuria continuously for eight days before operation and some others were no more favorable, some having had long existent chronic nephritis. Some who apparently would have recovered died from acute bronchitis, from pneumonia and one after twelve days from septicemia originating in the wound. Yet thirteen got well. It is too much to claim that renal decapsulation should be credited with all of these? We must agree that it did save many when nothing else could avail; 50 per cent. under these circumstances in the infancy of the operation. With just a fair chance, it will do much better.

With the beginning of eclampsia all accepted treatment should be carried through, the various remedies being used simultaneously as nearly as possible and the rest in rapid succession, the response to treatment being carefully noted. This will all be finished in six or eight hours and by that time if the condition is not satisfactory, if recovery is not reasonably certain, decapsulation should be done upon both kidneys. It should have been considered and arranged for before this to avoid delay by discussion with the family, or preparation for operation.

The following is a report of my own cases:

Case I.—Mrs. W., age 38 years, had had four normal labors; at term in fifth pregnancy; had had severe headaches, dimness of vision, epigastric pain and swelling of limbs for four to six weeks, but no medical attendance. Labor pains severe for about eighteen hours, then instrumental delivery at 6 p. m., August 15, 1907. Condition normal until first convulsion at 11.30 the same evening, passed directly into coma which persisted. At 11.30 next morning medicine, packs and sweating had been tried; she had grown worse all the time; had had fifteen or sixteen convulsions; pulse 180 to 200; very feeble, surface livid, capillary circulation very sluggish, pulmonary edema very marked. Temperature, 104.6 and rapidly rising (within the next hour it got to 105.4), urine by catheter, two ounces in six hours, loaded with albumen, various kinds of casts, blood and debris. At 12.30 noon, both kidneys were decapsulated. Both were deeply congested, one enlarged. There was one convulsion about an hour after the operation and no more. The temperature, edema and pulse regained normal in about forty-eight hours. Secretion of urine began to increase in about six hours, and in the twenty-four hours following the first twelve after operation amounted to over 100 ounces, besides what was passed with about eight stools themselves mostly water. This amount of urine continued several days. Consciousness began to appear thirty-six hours after operation, but was not complete until about five days. Repeated examinations recently show the urine to be normal in all respects. She is strong and well fourteen months after the operation.

Case II.—Mrs. E., age 36; third child. First labor normal; second preceded for about one month by albuminuria and swelling of the feet that improved under treatment. When about at term convulsions occurred without labor. Forced delivery was resorted to at once and she recovered rapidly. With the third pregnancy there were albuminuria and swelling of feet at the end of the seventh month, which again improved satisfactorily under treatment. Normal labor, August 11, 1908, at 11.30 p. m. Very soon she had agonising headache and vomiting, which persisted until the first convulsion at 9 a. m., August 12, immediately followed by coma, from which she never awakened. There were five convulsions in the first hour, no more till 3 a. m., August 13, when there was one, then several about 9 a. m. At this time the pulse was very weak and rapid and growing more so; temperature over 104 and rising, pulmonary edema present: marked anuria. Decapsulation of both kidneys at 11 a. m. Both very much enlarged and deeply congested. There was no improvement, no increase in amount of urine or lessening of coma or other symptoms. She died about 9.30 p. m. the same day.

BIBLIOGRAPHY.

Edebohls: N. Y. Med. Jour., 1903, LXXVII., p. 1022. I case. Recovered.

Edebohls: Boston M. and S. Jour., Vol. 150, 1904, p. 586. I case. Recovered.

Edebohls: Ctbl. f Gynækol, 1906, XXX., p. 719. I case. Recovered.

Chambrelent and Pousson: Bull. de 'l Acad. de med., 1906. Vol. LV., 489. 1 case. Reported by Pinard. Recovered.

Jardine: Jour. of Obstetrics and Gynecology, 1906, X., p. 32, 34. I case. Died.

De Bovis: La Semaine Medicale, Mar. 6, 1907. 1 case. Recovered.

Pieri: Annales de Gynecol. et d' Obstet. 1907, IV., p. 257. I case. Died.

Treub: Annales de Gynecol. et d' Obstet. 1907, IV., p. 314. 1 case. Died.

Gauss: Ctbl. f Gynækol, 1907, No. 19. 2 cases. Recovered.

Polano: Ctbl. f Gynækol, 1907, No. 1. 1 case. Recovered.

DeCotrel: Union Medicale du Canada, XXXVI., June 1, 1907. 1 case. Died.

O. Franck: Munch med. Wochenschr., Dec. 10, 1907. 1 case. Died.

Wiemer: Monatschrift f Geburtshulfe u Gynækolie, 1908, XXVIII., p. 321. 3 cases. First case, recovered; second case, died; third case. recovered.

Asch: Abl. f Gynækol, 1908. No. 2. 1 case. Recovered. Reported by Falgowski.

Asch: Ctbl. f Gynækol, 1908. No. 9. 1 case. Recovered.

Kleinertz: Ctbl. f Gynækolokie, 1908. No. 26. Two cases. First case, died; second case, recovered.

Essen-Moller: Ctbl. f Gynækologie. No. 14. 1908. 1 case. Died

Hamin: Ctbl. f Gynækologie, 1908. No. 20. 1 case. Died.

